# Mental toughness in adolescents: bridging family relationships and depression across personality traits

**DOI:** 10.1186/s40359-024-01702-z

**Published:** 2024-04-17

**Authors:** Feirui Ni, Yawen Zheng, Sheng Qian, Guanghui Shen, Wen-jing Yan, Yu-Wei Wu, Ziye Huang

**Affiliations:** 1 Wenzhou Seventh People’s Hospital, 325035 Wenzhou, China; 2 Lishui Second People’s Hospital, Wenzhou Medical University, Lishui, China; 3 The Affiliated Wenzhou Kangning Hospital, Wenzhou Medical University, Wenzhou, China; 4 Wenzhou Medical University, Wenzhou, China; 5 Student Affairs Division, Wenzhou Business College, 325035 Wenzhou, China

**Keywords:** Adolescents, Family relationships, Mental toughness, Personality traits, Mental health

## Abstract

**Background:**

Adolescence is a pivotal stage vulnerable to mental health issues like anxiety and depression. While family relationships, mental toughness, and personality traits are known to impact adolescent mental health, their interactive and moderating roles are not fully understood.

**Aim:**

This study aims to investigate the mediating role of mental toughness in the relationship between family relationships and depression among high school students, and to examine the varying impacts of personality traits on this mediation.

**Method:**

A cross-sectional study was conducted on a sample of 734 adolescents. Participants completed measures assessing family relationships, mental toughness, personality traits, and mental health outcomes (depression). Latent Profile Analysis, Multiple Regression Analysis, and Structural Equation Modeling, to investigate these relationships.

**Results:**

The study found that mental toughness significantly mediates the relationship between family relationships and depression. Notably, this mediating effect varied between personality type; it was more pronounced in the moderate-reserved type compared to the proactive-engaged type. LPA identified two distinct personality types of students based on their personality traits, with differential patterns of family relationships, mental toughness, and depression. Multiple regression analysis indicated that character and adaptability, components of mental toughness, were significant negative predictors of depression.

**Conclusion:**

The study contributes to understanding the dynamics of adolescent mental health, particularly in the context of Chinese high school students. It underscores the importance of considering family dynamics, personality traits, and mental toughness in developing effective mental health interventions for adolescents.

## Introduction

In the realm of adolescent mental health, understanding the intricate interplay between familial dynamics, personality traits, and mental resilience remains a cornerstone of psychological research. The impact of family relationships on the psychological well-being of adolescents has long been acknowledged in the field, underscoring the significance of the familial environment in shaping emotional and psychological development during these formative years [1, 2]. Concurrently, the role of personality traits has garnered attention for its profound influence on coping mechanisms and mental health outcomes in adolescents [3]. These two strands of research converge in exploring the vulnerabilities and strengths that emerge during adolescence, a period marked by significant psychological and emotional changes.

However, despite considerable advancements in understanding these dynamics, the concept of mental toughness, especially as a mediating factor between family relationships and mental health outcomes, has not been comprehensively explored. Mental toughness, a construct associated with resilience, adaptability, and psychological endurance, is increasingly recognized as a pivotal element in adolescent psychological health [4]. It represents a blend of emotional and cognitive capabilities that enable individuals to navigate challenges and stressors effectively. The exploration of how mental toughness interacts with family dynamics and personality traits in shaping mental health outcomes, particularly depression, in adolescents is crucial for developing targeted interventions and support mechanisms.

### Several related factors to adolescents’ depression

The existing body of literature underscores the pivotal role of family relationships in adolescent mental health. Research has consistently highlighted how positive family dynamics can act as a protective factor against various psychological disorders, including depression [5, 6]. Family warmth, cohesion, and supportive parenting practices are associated with better mental health outcomes and lower levels of depressive symptoms in adolescents [7]. On the contrary, familial conflict and dysfunction have been linked to increased risks of depression and other mental health challenges [8].

In parallel, the influence of personality traits, particularly extraversion and introversion, on adolescent mental health has been extensively studied. Extraversion has been positively associated with better mental health and well-being, attributed to extroverts’ propensity for social engagement and positive affect [9]. Introverted adolescents, conversely, have shown a higher prevalence of internalizing problems, including anxiety and depression [10]. However, the understanding of these traits is not unidimensional, as the interaction of these personality traits with environmental factors, such as family relationships, is less understood.

Mental toughness, a concept that has gained traction more recently, is increasingly being recognized for its role in adolescent mental health. It encompasses elements of resilience, coping skills, and emotional regulation [11]. Studies have begun to explore its protective role against stress and psychological difficulties, suggesting that mentally tough adolescents are better equipped to handle stressors and challenges [12]. However, the literature is still evolving in terms of understanding the intricate ways in which mental toughness interacts with family dynamics and personality traits.

When exploring the relationship between family relationships, personality traits and adolescent depression, researchers paid special attention to the mediating role of mental toughness and the moderating role of personality traits. Research shows that supportive family relationships and positive interactions can enhance adolescents’ mental resilience, thereby reducing the incidence of depressive symptoms [13]. In addition, personality traits, such as extraversion versus introversion, may moderate the strength of this mediating effect, in which extroverted adolescents may show stronger mental resilience in the face of stress due to their higher social participation and positive affectivity [14]. Although research has examined the relationship between these variables and adolescent depression, the specific mechanisms underlying the mediating role of mental toughness and the moderating role of personality traits are still unclear.

Despite these significant advancements in understanding these separate strands, the existing research falls short in comprehensively exploring how these factors interact in a more integrated manner. Particularly, the mediating role of mental toughness between family relationships and mental health outcomes, in the context of different personality traits, remains an underexplored area. This gap is critical as it hinders the development of more nuanced interventions and support systems for adolescents struggling with mental health issues. The potential interplay of these factors could offer insights into the mechanisms through which family environments and personality traits influence mental health through the lens of mental toughness.

### The gaps between the literature and the current study

Building on the existing research, several critical gaps become evident. Firstly, while the influence of family relationships and personality traits on adolescent mental health is well-documented [15, 16], their interaction and combined impact on mental health outcomes, particularly through the mediating role of mental toughness, remain underexplored. The majority of studies have treated these elements in isolation, overlooking the potential synergistic or counteractive effects they might have in conjunction with each other. This oversight presents a significant gap in the literature, as the interplay between family dynamics, personality traits, and mental toughness could offer a more comprehensive understanding of the factors contributing to or mitigating against adolescent depression.

Furthermore, the nuanced differences between personality types in relation to mental toughness and family relationships, have not been adequately addressed in the context of mental health outcomes. This gap suggests a lack of understanding of how personality traits might influence or alter the impact of family relationships and mental toughness on mental health. Given the distinct ways in which extraverts and introverts process social interactions and stressors, it is crucial to explore how these personality types differently mediate the relationship between family dynamics, mental toughness, and depression.

Therefore, this study aims to address these gaps by investigating the mediating role of mental toughness in the relationship between family relationships and depression among high school students, while also examining the differential effects of personality types on this mediation. The objectives of this study are twofold:

1. To assess the extent to which mental toughness mediates the relationship between family relationships and depression in high school students.
2. To explore how this mediation is influenced by the personality type.

## Methods

### Participants

This study was conducted with a census of high school students from Wenzhou. Participants were screened based on specific inclusion criteria to ensure the integrity and quality of the data. We excluded individuals with diagnosed dyslexia or other reading disorders, as well as those who declined to sign the informed consent form. To further enhance the reliability of our data, instructional manipulation checks were embedded within the assessment to identify and exclude random or insincere responses. Out of the initial 923 participants who began the study, 189 were eliminated based on their responses to the instructional manipulation checks. Total 734 students who provided valid and usable data, resulting in an effective response rate of 79.52%. The sample comprised 439 males (59.81%) and 295 females (40.19%).

### Procedure

Upon receiving the school administration’s approval and adhering to ethical guidelines, a detailed procedure was established to facilitate the data collection process. Participation was voluntary, and anonymity was guaranteed to all participants to encourage honest responses. Prior to data collection, informed consent forms were distributed and signed by the participants, ensuring they were fully aware of the study’s purpose and their rights as participants. The data collection was conducted in a controlled environment to minimize distractions and potential confounding variables. A trained professional, certified in psychological education, led the participants to the school’s computer room designated for the assessment. Ethical adherence was maintained throughout, conforming to national and institutional human experimentation standards and the Declaration of Helsinki (revised 2008), with approval by the IRB of the Seventh People’s Hospital of Wenzhou (EC-KY-2,022,048).

### Measurements

#### Demographic information

Demographic information was collected from participants through self-report questions. Gender was measured using a single self-coding question, with 1 representing male and 2 representing female. Family relationships were assessed using three self-administered questions rated on a 5-point Likert scale ranging from 1 (Very poor) to 5 (Very good): “How warm I think the family atmosphere is”, “My relationship with my parents”, and “The relationship between my parents”. The family relationship total score was calculated as the sum of responses to these three questions, with higher scores indicating better family relationships (possible range 3–15). This brief 3-item family relationships scale demonstrated good internal consistency reliability in the present study (Cronbach’s alpha α = 0.79).

#### Personality trait

Personality traits were measured using the 10-item Personality Inventory (TIPI) scale. The TIPI is a brief measure developed designed to assess the Big Five personality dimensions (extraversion, agreeableness, conscientiousness, emotional stability, and openness to experiences) [17]. It includes 10 items, with 2 items representing each dimension rated on a 6-point scale. The TIPI has demonstrated adequate psychometric properties in China, with Cronbach’s α = 0.79 and the split-half reliability of the Spearman–Brown coefficient was 0.51 [18].

#### Mental toughness

Mental toughness was assessed using the Brief Mental Health Diathesis Scale developed. The scale consists of 36 items and measures three dimensions of Chinese mental toughness, including cognition, character and adaptability. Participants rated items on a 5-point Likert scale ranging from 1 (not at all consistent) to 5 (completely consistent), with higher scores reflecting greater mental toughness. This scale has shown good reliability and validity in Chinese samples, with Cronbach’s α = 0.89 [19].

#### Depression

Depressive symptoms were assessed using the Center for Epidemiological Studies Depression Scale (CES-D) [20]. The CES-D is a well-established instrument consisting of 20 items in which participants are asked to indicate how often they experienced depression-related symptoms during the past week. Responses are rated on a four-point Likert scale ranging from 1 (rarely or never) to 4 (most or all of the time), with higher scores indicating greater depressive symptoms. In the current study, the CES-D demonstrated strong internal consistency with Cronbach’s alpha α = 0.92.

### Statistics analysis

Initially, we conducted a descriptive analysis to characterize the basic demographics and variable distributions within the participants. Measures of mean and standard deviation were calculated for all continuous variables, while frequencies and percentages were reported for categorical variables and dummy variable conversion was performed for categorical variables. Subsequently, Pearson’s bivariate correlation analysis was performed to evaluate the correlation among the variables, including gender, family relationships, cognition, character, adaptability, and depression. The threshold for statistical significance was set at *p* < 0.05. To identify potential subgroups within the participants, latent profile analysis (LPA) was employed using a robust maximum likelihood estimator [21]. Model selection was guided by several fit indices: the Akaike Information Criterion (AIC), the Bayesian Information Criterion (BIC), and the Bootstrap Likelihood Ratio Test (BLRT), with lower values indicating better model fit [22]. Entropy values close to 1 suggested clearer delineation of classes. The decision on the number of profiles was based not only on statistical criteria but also on the interpretability and theoretical justification of the identified classes. Based on the results of the LPA, a new variable called “Personality Type” was created to represent the identified potential subgroups.

Then, multiple regression analysis was conducted to examine the predictive power of the identified variables on depression scores. The regression included main effects and interaction terms to evaluate whether family relationships, mental toughness and personality type had interactive effects on depression beyond their main effects. The variables were mean-centered to mitigate multicollinearity prior to constructing interaction terms [23]. Standardized beta coefficients (*β*) were reported to assess the strength of the predictors.

Finally, to further elucidate the relationships between family relationships, mental toughness, and depression, Structural Equation Modeling (SEM) was implemented. SEM allowed for the examination of both direct and indirect paths, providing insight into potential mediation effects. The SEM analysis was stratified by the subgroups identified through LPA to ascertain whether patterns differed between them. Goodness-of-fit indices, including the Chi-square statistic (χ2), the Comparative Fit Index (CFI), and the Root Mean Square Error of Approximation (RMSEA), were reported to evaluate model fit. Indirect effects were tested via bootstrapping methods, using 1000 bootstrap samples with bias-corrected confidence intervals to ascertain significance.

All analyses were conducted using R statistical software. LPA was implemented with the “mclust” package, regression analyzed using the base “stats” package, and SEM performed with the “lavaan” package. Effects were considered statistically significant at *p* < 0.05.

## Result

### Descriptive statistics

In a sample comprising 734 participants, 439(59.81%) were male and 295(40.19%) were female. Table 1 presents the results of correlation analyses and descriptive statistics for several variables. The result reveal that gender did not show any significant correlation with the other examined variables. Family relationships showed significant correlation with various factors: there was a positive correlation with cognition (*r* = 0.37, *p* < 0.001), character (*r* = 0.36, *p <* 0.001), and adaptability (*r* = 0.44, *p* < 0.001), a negative correlation with depression (*r* = -0.40, *p* < 0.001). Further, cognition, character and adaptability were significant correlated with depression (*p* < 0.001).

**Table 1:** Descriptive statistics and correlation analysis (*N* = 734)

Variable	1	2	3	4	5	6
1. Gender	1					
2. Family Relationships	0.01	1				
3. Cognition	-0.05	0.37^***^	1			
4. Character	0.04	0.36^***^	0.47^***^	1		
5. Adaptability	-0.01	0.44^***^	0.74^***^	0.68^***^	1	
6. Depression	0.03	-0.40^***^	-0.48^***^	-0.73^***^	-0.67^***^	1
*M*	(--)	11.83	32.04	30.67	61.04	37.51
*SD*	(--)	1.96	7.44	6.73	11.62	9.71

Note.^***^p < 0.001; The “Personality Type” variable, generated through latent profile analysis in Sect. 3.2, is not included in this basic variable report

### Latent profile analysis

LPA was conducted to discern potential classes within the data. Table 2 illustrates the fitting metrics for models ranging from one to seven classes. Several criteria, such as the AIC, BIC, and BLRT, were utilized to evaluate model fit. Additionally, entropy values, indicative of classification accuracy, and the relative sizes of the smallest (N_min) and largest (N_max) classes were considered to avoid class solutions with excessively small or large classes. Notably, as the number of classes increased from one to seven, there was a consistent decline in both AIC and BIC values, suggesting improved fit with the inclusion of additional classes. However, the model with two classes (Model 2) showed highest entropy value of 0.86. The relative sizes of the smallest and largest classes for this model were 0.23 and 0.77, respectively. Given the convergence of evidence from the Entropy value and N_min, Model 2 was ultimately chosen as the most suitable representation of the data.

**Fig. 1:**
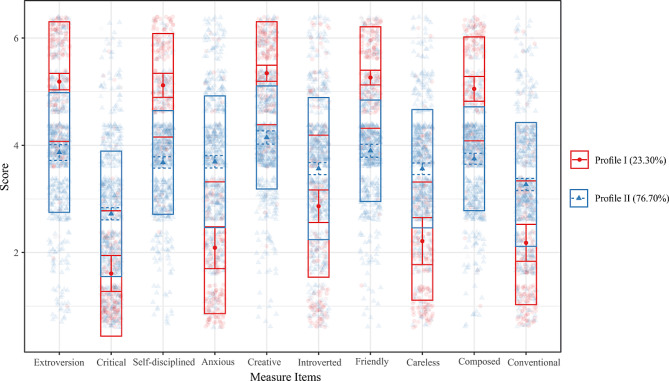
Scatter plot of the 2-Class LPA model Note: The 10 dimensions on the X-axis of Fig. 1 correspond to the 10 items of the TIPI cale. The order of the dimensions on the X-axis follows the sequence of items in the TIPI scale. The dots in each box denote the median scores of the individuals and the vertical spread of each box represents the interquartile range of scores

The mean scores of the two classes across different measurement items are depicted in Fig. 1. Class 1, labeled the “Extraversion Group,” encompasses 171 students (23.30%) and is typified by proactive, outgoing, and energetic characteristics. Conversely, Class 2, labeled the “Introversion Group,” comprises 563 students (76.70%), who generally appear to be introverted and less proactive. These profiles were then used to create a dummy variable called ‘Personality Type’ with participants assigned a value of 0 for the Proactive-Engaged Type (Profile I) and 1 for the Moderate-Reserved Type (Profile II).

Further analyses compared the two identified subgroups, proactive-engaged and moderate-reserved, across several domains: family relationships, mental toughness, and depression. As showed in Table 3, The proactive-engaged type had significantly higher family relationship scores than the moderate-reserved type (*p* < 0.001), with *Cohen’s d* = 0.76. For the broader dimension of mental toughness, the proactive-engaged type showed higher cognition, character, and adaptability than the moderate-reserved type (*p* < 0.001), especially adaptability, which showed the largest effect size in this study with *Cohen’s d* = 1.40.

**Table 2:** Fitting information for Class 1–7 LPA models

Model	AIC	BIC	Entropy	N_min	N_max	BLRT(p)
1	23757.42	23849.39				
2	22764.95	22907.50	0.86	0.23	0.77	0.01
3	22503.89	22697.02	0.72	0.16	0.51	0.01
4	22451.01	22694.73	0.75	0.08	0.49	0.01
5	22232.75	22527.05	0.81	0.02	0.57	0.01
6	22093.91	22438.80	0.83	0.04	0.55	0.01
7	22038.21	22433.68	0.80	0.02	0.49	0.01

**Table 3:** The comparison between profiles

Variables	Proactive-Engaged Type (Profile I)	Moderate-Reserved Type (Profile II)	t	*p*	Cohen’s d
Family relationships	12.91 ± 1.70	11.50 ± 1.91	8.68	< 0.001	0.76
Depression	30.01 ± 7.76	39.79 ± 9.08	12.73	< 0.001	1.11
Mental toughness					
Cognition	38.22 ± 6.25	30.17 ± 6.73	13.93	< 0.001	1.22
Character	35.83 ± 6.03	29.11 ± 6.12	12.62	< 0.001	1.10
Adaptability	71.80 ± 10.42	57.77 ± 9.86	16.08	< 0.001	1.40

### Multiple regression analysis

A multiple regression analysis was conducted to examine the predictors of depression (refer to Table 4). The overall regression model was statistically significant (*F*_(8, 725)_ = 114.46, *p* < 0.001), and accounted for 60.70% of the variance in depression scores (*Adjusted-R*^2^ = 0.61). The result reveal that personality type was a significant positively associated with depression (*β* = 0.11, *p* < 0.05) after controlling for other variables. In mental toughness, character and adaptability were significant negatively associated with depression, suggesting that higher character and adaptability scores were associated with lower depression (*p* < 0.05). Apart from this, the interaction terms between personality type and family relationship, cognition, character, and adaptability were not statistically significant (all *p*s > 0.05). It is notable that family relationship was significantly correlated with depression in the bivariate correlation analysis but did not emerge significant association within the multiple regression model (*β* = -0.09, *p* > 0.05). This suggests that family relationships may have overlapped variance with other factors and that the association family relationship is diminished in the context of other variables.

**Table 4:** Multiple regression analysis examining the predictors of depression

	Variables	β	SE	t	*P*	ΔR^2^	F
Step 1						< 0.001	0.48
	Gender	0.04	0.02	1.63	0.10		
Step 2						0.60	187.11^***^
	Personality	0.11	0.03	3.15	0.002		
	Family Relationship	-0.09	0.06	1.56	0.12		
	Mental Toughness						
	Cognition	0.12	0.08	1.51	0.13		
	Character	-0.44	0.07	6.69	< 0.001		
	Adaptability	-0.25	0.08	3.01	0.003		
Step 3						0.006	114.46^***^
	Family Relationship × Personality Type	-0.08	0.07	1.17	0.24		
	Cognition × Personality Type	-0.06	0.06	1.07	0.29		
	Character × Personality Type	-0.06	0.07	0.78	0.44		
	Adaptability × Personality Type	0.01	0.06	0.10	0.92		

### Structural equation model

**Fig. 2:**
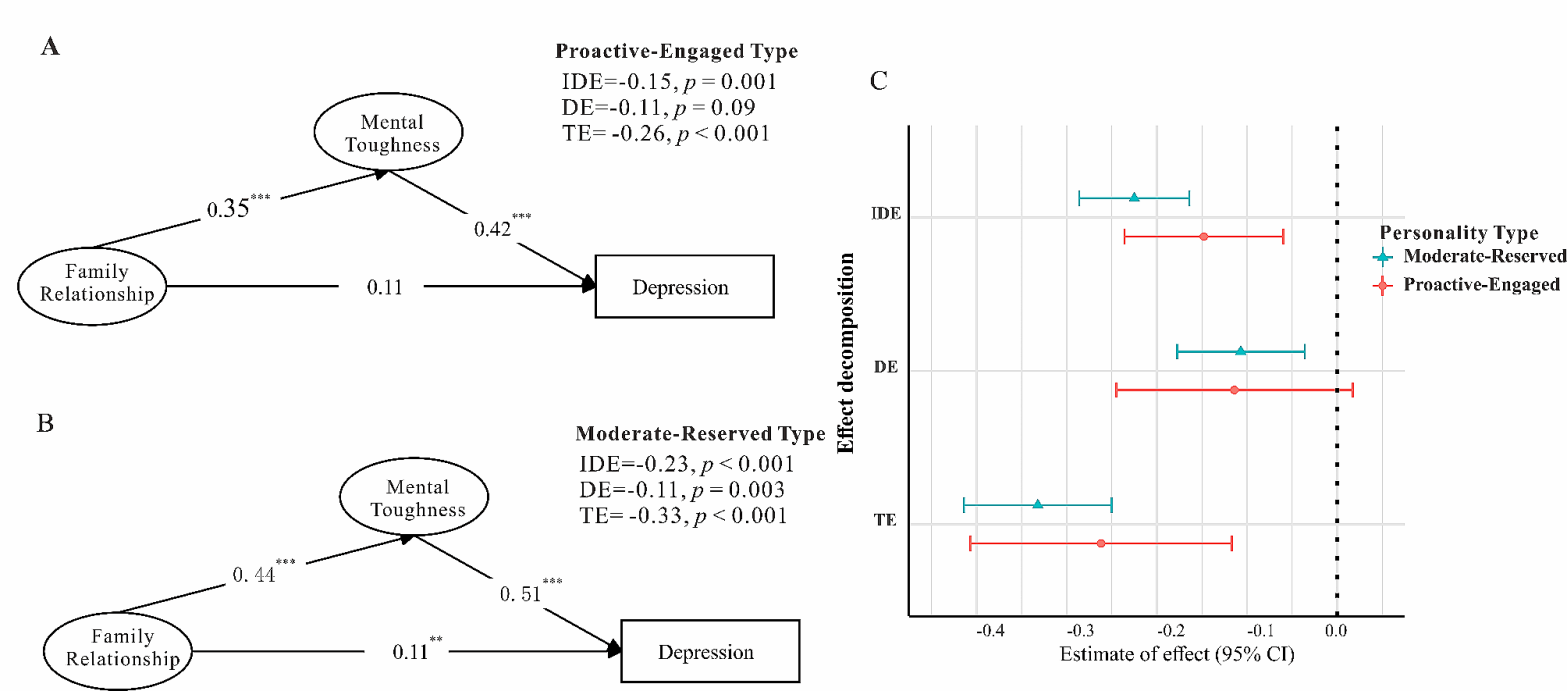
Mediation model and effect decomposition by personality Note: (**A**) Model for proactive-engaged type showing mental toughness fully mediating the relationship between family relationships and depression. (**B**) Model for moderate-reserved type showing partial mediation, with a significant direct effect remaining. (**C**) Decomposition of total effect into direct and indirect (through mental toughness) components. The indirect effect is significant in both groups, but the direct effect only remains significant in the moderate-reserved type

Given the pattern of findings showing an association between family relationships and depression that disappeared when controlling for other variables, structural equation modeling was employed to test mental toughness as a potential mediating mechanism. This approach allowed modeling the direct and indirect effects simultaneously in order to explicate the relationships between these variables. Structural equation modeling was utilized to test mental toughness as a mediator between family relationships and depression separately for the proactive-engaged and moderate-reserved type. Model fit indices for both types were adequate, indicating the data fit the hypothesized model well (extraversion group: χ^2^_(11)_ = 16.701, *CFI* = 0.99, *RMSEA* = 0.06; introversion group: χ^2^_(11)_ = 20.601, *CFI* = 0.99, *RMSEA* = 0.04). For the proactive-engaged type, the path from family relationships to mental toughness was significant (*β* = 0.35, *p* < 0.001), as was the path from mental toughness to depression (*β* = -0.42, *p* < 0.001). The direct path from family relationships to depression was non-significant (*β* = -0.11, *p* > 0.05). A similar pattern emerged for the moderate-reserved type, with significant paths from family relationships to mental toughness (*β* = 0.44, *p* < 0.001) and from mental toughness to depression (*β* = -0.51, *p* < 0.001). However, unlike the proactive-engaged type, the direct path from family relationships to depression was significant (*β* = -0.11, *p* < 0.01), suggesting partial mediation. The results of the effect decomposition and more details was showed in Fig. 2.

## Discussion

The primary purpose of our study was to explore the intricate and dynamic relationship between family relationships, mental toughness, depression, and personality traits in high school students. Our study reveals several important findings that give us a nuanced understanding of the dynamics of adolescent mental health. First, we found a strong correlation between positive family relationships and higher scores on the mental toughness dimensions. Furthermore, we found that higher levels of mental toughness were inversely associated with depressive symptoms. This suggests that mental toughness can act as a protective factor for depression, highlighting its potential as a target for psychological interventions [24]. Interestingly, our analyses also revealed that personality traits, particularly those associated with extraversion and introversion, had a significant impact on the relationship between family relationship, mental toughness, and depression. Furthermore, by highlighting the differential impact of these factors on extroverted and introverted individuals, our study adds a new dimension to understanding the role of personality in mental health. This insight is critical for the development of mental health interventions that take into account individual personality differences. These findings fill an important knowledge gap in the field of adolescent psychology. Previously, the interactions between family environment, personality traits, and mental health outcomes were poorly understood, especially in the context of Chinese high school students. Our study not only reveals these relationships, but also provides empirical evidence that parenting in the family environment and the development of mental toughness are key factors influencing adolescents’ mental health.

### Comparative analysis of mental toughness and depression between personality type

Personality type have long been studied in relation to mental health outcomes. Proactive engaged type is characterized by sociability, assertiveness, and positive emotionality, while moderate-reserved type is marked by a preference for solitude and internal experiences. Our findings suggest that proactive engaged adolescents exhibit higher levels of mental toughness and lower levels of depression. This aligns with studies suggesting proactive engaged may possess better social support networks due to their sociable nature, which can be a buffer against mental health challenges [25]. Additionally, proactive engaged has been linked to positive affectivity [26], which can promote mental toughness and coping skills, key components of mental toughness.

Conversely, moderate-reserved individuals in our study showed lower mental toughness and higher depression rates. This finding is consistent with research indicating that introverts are more likely to engage in rumination, a risk factor for depression [27]. Their tendency to internalize experiences may also limit their exposure to external coping resources, potentially leading to lower mental toughness.

Mental toughness, comprising factors like cognition, character, and adaptability, plays a differential role across personality types [28]. For proactive engaged type, their inherent tendency towards positive emotionality and sociability may naturally reinforce the elements of mental toughness. In contrast, moderate-reserved type might need to develop these aspects more consciously, as their natural predisposition may not lend itself easily to traits like adaptability.

From a theoretical perspective, these findings emphasize the necessity of considering personality traits when examining the constructs of mental toughness and depression. Clinically, this suggests that interventions aimed at enhancing mental toughness and mitigating depression in adolescents need to be tailored according to personality types [29]. For instance, strategies that work for proactive engaged individuals, such as group-based therapies or activities that leverage their sociability, may not be as effective for moderate-reserved individuals, who may benefit more from individualized approaches that focus on internal coping mechanisms.

### Influence of family relationship on mental toughness

The relationship between family relationship and mental toughness, as observed in our study, extends beyond a mere correlation. It suggests a formative role of familial interactions in shaping an individual’s mental toughness. This idea resonates with the ecological systems theory proposed by Bronfenbrenner [30], which posits that an individual’s development is deeply influenced by their interactions with various environmental systems, with the family being a primary microsystem. Our findings that positive family relationships correlate with higher mental toughness can be interpreted through the lens of attachment theory [31, 32]. This theoretical interpretation suggests a need for interventions focusing on strengthening family bonds as a strategy for enhancing mental toughness in adolescents.

### The mediating effect of mental toughness between the family relationships and depression: differential effects across personality types

A key finding of this study was the significant mediating effect of mental toughness in the relationship between family relationships and depression. This result suggests that positive family relationships may foster the development of mental toughness, which in turn acts as a protective factor against depressive symptoms in adolescents. Our findings align with previous research highlighting the importance of family support and cohesion in promoting resilience and mental well-being in young people [33, 34]. However, our study extends this literature by demonstrating the specific mediating role of mental toughness, providing a clearer understanding of the mechanisms linking family factors to adolescent mental health outcomes. Notably, we found that the mediating effect of mental toughness differed between the two personality types identified through latent profile analysis: the proactive-engaged type and the moderate-reserved type. This medicating effect is fully mediated in the proactive-engaged type, but partially mediated in the moderate-reserved type. This indicates that for moderate-reserved type family relationship is an independent important protective factor in addition to the individual’s mental toughness. This study highlights the significance of accounting for individual differences when investigating the routes to mental health. While the mediating role of mental toughness was the primary focus of this study, our results also shed light on the influence of family relationships on mental toughness itself. We found that more positive family relationships were associated with higher levels of mental toughness, consistent with previous research suggesting that supportive family environments can foster resilience and coping skills in adolescents [35]. This finding highlights the potential for family-based interventions to promote the development of mental toughness, which may in turn buffer against the onset of depressive symptoms.

### Limitations and future implications

The interpretation of our study’s findings must be considered in light of several limitations. Firstly, the sample was exclusively drawn from high school students in Wenzhou, which may limit the generalizability of the results. As noted in cross-cultural psychology research, cultural factors significantly influence psychological constructs [36, 37]. The regional specificity of the sample could have influenced the observed relationships between family relationship, mental toughness, and depression. This raises concerns about the potential impact of response biases on the study’s findings. The use of self-report measures, despite being a standard practice in psychological research, could have led to skewed interpretations of family relationship, personality traits, or symptoms of depression [38]. This is particularly relevant considering the age group of the participants, as adolescents might have varying levels of self-awareness and willingness to report sensitive information accurately. These limitations do not invalidate the study’s findings but rather provide a context for their interpretation. They highlight the need for caution in extending these results to broader populations without considering cultural and contextual differences. Additionally, they underscore the importance of using diverse methodological approaches, including objective measures and multi-informant reports, in future research to corroborate and expand upon these findings. Future studies should aim to include a more diverse and geographically varied sample to enhance the generalizability of the findings [39, 40]. Additionally, incorporating alternative assessment methods, such as behavioral observations or informant reports, could provide a more holistic understanding of the constructs being studied.

## Data Availability

The data that support the findings of this study are available from the corresponding author upon reasonable request.
